# Prospective identification of parasitic sequences in phage display screens

**DOI:** 10.1093/nar/gkt1104

**Published:** 2013-11-09

**Authors:** Wadim L. Matochko, S. Cory Li, Sindy K.Y. Tang, Ratmir Derda

**Affiliations:** ^1^Department of Chemistry and Alberta Glycomics Centre, University of Alberta, Edmonton, AB T6G 2G2, Canada, ^2^Department of Bioengineering, MIT, Cambridge, MA 02139, USA and ^3^Department of Mechanical Engineering, Stanford University, Stanford, CA 94305, USA

## Abstract

Phage display empowered the development of proteins with new function and ligands for clinically relevant targets. In this report, we use next-generation sequencing to analyze phage-displayed libraries and uncover a strong bias induced by amplification preferences of phage in bacteria. This bias favors fast-growing sequences that collectively constitute <0.01% of the available diversity. Specifically, a library of 10^9^ random 7-mer peptides (Ph.D.-7) includes a few thousand sequences that grow quickly (the ‘parasites’), which are the sequences that are typically identified in phage display screens published to date. A similar collapse was observed in other libraries. Using Illumina and Ion Torrent sequencing and multiple biological replicates of amplification of Ph.D.-7 library, we identified a focused population of 770 ‘parasites’. In all, 197 sequences from this population have been identified in literature reports that used Ph.D.-7 library. Many of these enriched sequences have confirmed function (e.g. target binding capacity). The bias in the literature, thus, can be viewed as a selection with two different selection pressures: (i) target-binding selection, and (ii) amplification-induced selection. Enrichment of parasitic sequences could be minimized if amplification bias is removed. Here, we demonstrate that emulsion amplification in libraries of ∼10^6^ diverse clones prevents the biased selection of parasitic clones.

## INTRODUCTION

**Figure 1.**
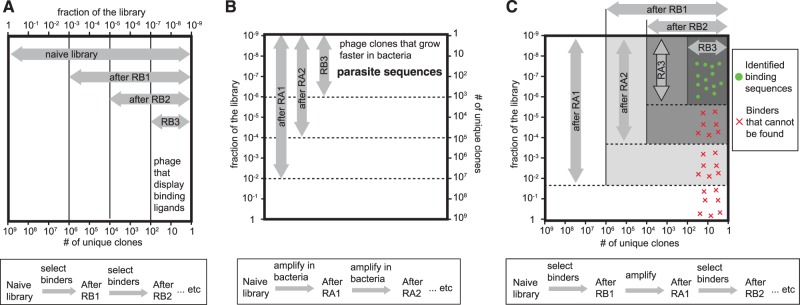
(**A**) Selection from phage display libraries after rounds of binding (RB) to the target can be represented as progressive collapse of naïve library (10^9^ diverse sequences) to a smaller number of binding sequences (here, 10^2^ sequences). (**B**) It is known that the naïve library of phage-displayed peptides contains sequences that amplify slowly in bacteria and those that amplify faster. Repetitive rounds of amplification (RA) in bacteria, thus, lead to progressive collapse of diversity from the theoretical 10^9^ clones to a smaller number of binding sequences. (**C**) Collapse due to binding preferences and due to amplification in bacteria are independent of one another. In a selection that involves rounds of binding and re-amplification, library collapses to a few clones that bind to a target and have high amplification rates. As a consequence, many binders, labeled as ‘x’, cannot be discovered in conventional phage display selection.

*In vitro* evolution and selection of genetic libraries is central to molecular biology research. In drug discovery, the selection of lead compounds from random genetically encoded libraries complements rational drug design. Many Food and Drug Administration (FDA)-approved antibodies and peptides on the market have originated from selection and evolution experiments (1,2). Selection from genetically encoded libraries is finding increasing utility in areas such as the development of new chemicals, the design of new materials and the discovery of new chemical reactions (3–5). Screening experiments—such as phage display (6,7), nucleotide display, cell display, Systematic Evolution of Ligands by Exponential Enrichment (SELEX) and DNA aptamer selection (8,9)—use libraries with a diversity of >10^9^ unique sequences, which are then narrowed to 10^2^–10^6^ useful library members. In a screen that aims to identify binding sequences for a specific target, selection increases the abundances of sequences that have high binding capacity. Sequencing of clones enriched during *in vitro* selection is often used to analyze the selection preferences and the enrichment for sequence motif(s). Collapse of the naïve library to a collection of a few sequences indicates that selection narrowed onto clones that bind to a target (Figure 1A). While most screens exhibit convergence to one sequence motif, screens against the surfaces of cells or tissues (10–12), mixtures of antibodies (13–16) or other proteins, could converge on multiple binding epitopes. The screens against such ‘multisite targets’ could yield information about multiple ligands for multiple receptors on the cell (10,11). In recent years, deep sequencing approaches have been used to assist the analysis of phage-displayed selection (17), and in many cases, the selection against multisite targets (18–20). Our group used deep sequencing to detect convergence, which occurs in the phage display screens without any selection (Figure 1B). We amplified 10^6^ sequences from a naïve library in bacteria, and observed that amplification alone enriched a few hundred motifs by 10–100-fold and depressed the remaining 10^6^ motifs (21). This experiment, for the first time quantified the collapse of the library during amplification in bacteria in the absence of any target-driven selection. It is possible that in screening for some targets, biological factors that favor amplification might also favor target binding (22). For many targets, amplification-induced collapse is largely independent from the collapse induced by target-binding selection (23). A typical phage display procedure that contains multiple rounds of target-binding (panning) and amplification in bacteria is thus driven by two separate selection pressures (Figure 1C). There are two fundamental predictions from Figure 1C: (i) selection could identify only a small number of available binding clones (green dots in Figure 1C); (ii) most of the selections should co-cluster with fast-growing clones, which from here on are referred to as ‘parasitic clones’. Figure 1C is a theoretical prediction (23), which we confirm in this report.

There is numerous evidence in the literature that enrichment of sequences in phage display is driven by two pressures: (i) affinity of binding to target; (ii) rate of amplification in bacteria. First reports of bias induced by amplification in bacteria (24) appeared in the phage literature a few years after the original description of peptide libraries (6,25,26). This bias was characterized extensively in libraries displayed on major coat protein pVIII (27–31). Makowski and coworkers quantified bias in pIII-displayed libraries (22,32), and used this information to develop a predictor of sequence-specific censorship (33). Periplasmic export of phage proteins through the *Sec* pathway, in general, was found to be a detriment to the display of globular proteins; this bias could be overcome by switching from *Sec* to other export pathways (34) [for an in-depth review of mechanistic origin of bias see (35)]. Finally, sequence-independent bias caused by mutations in regulatory regions of the phage genome has been uncovered by research groups of Smith and Noren (36,37). The effect these biases have on a library-wide scale was not known until recently (21).

Despite abundant information about amplification-induced bias, it is often viewed as an experimental inconvenience that could be overcome by improvements in the target-binding procedure (e.g. more washing steps). In this article, we show that amplification-induced bias is ubiquitous in phage display screens during the amplification of the Ph.D.-7, Ph.D.-12 and Ph.D.-C7C libraries. Parasitic or fast-growing clones are abundant in the naïve libraries. There are only two general strategies to avoid the bias: (i) avoid any amplification; (ii) use amplification that enriches all phage clones uniformly (38,39). In this report, we confirm that the latter strategy can remove sequence bias and avoid enrichment of parasitic clones.

## MATERIALS AND METHODS

### Phage libraries and their amplification

All libraries used in this report were purchased from New England Biolabs (NEB). Lot numbers were Ph.D.-7 (# 0061101), Ph.D.-12 (# 0101002) and Ph.D.-C7C (# 3). Reported diversity for each library was 10^9^ sequences. For amplification, we used *Escherichia coli* ER2738, which was maintained on solid media with tetracycline (Tet) as recommended by NEB. We prepared an overnight culture (ca. 10^9^ cfu/ml) from a single colony, and before phage amplification, we diluted it 1:100 with fresh Lysogeny Broth (LB) medium to yield ca. 10^7^ cfu/ml (‘cfu’ stands for ‘colony-forming units’). Each library was amplified under three different conditions:

Condition 1, bulk amplification of a 10^6^ subset of the library: 10^6^ plaque-forming units (PFU) from the naïve library were mixed with 10^7^ cfu of *E. coli* in 1 ml of LB. The mixture was shaken at 200 rpm for 5 h at 37°C. Amplification increased the titer from 106 to 1012 PFU; on average, each library clone should be amplified by a factor of 10^6^. Single-stranded DNA (ssDNA) was isolated from 10^11^ PFU.
Condition 2, bulk amplification of the entire naïve library: 10^9^ PFU from the naïve library were mixed with 10^10^ cfu of *E. coli* in 1 l of LB. The mixture was shaken at 200 rpm for 5 h at 37°C. Amplification increased the titer from 10^9^ to 10^15^ PFU; on average, each library clone should be amplified by a factor of 10^6^. ssDNA was isolated from 10^12^ PFU.
Condition 3, emulsion amplification of a 10^6^ subset of the library: 10^6^ PFU from the naïve library were mixed with 10^7^ cfu of *E. coli* in 3 ml of LB and emulsified using a microdroplet generator as previously described (38). The microdroplet generator produces ∼4 × 10^5^ droplets/ml; 3 ml of LB was used to ensure each clone was encapsulated into individual compartments and to avoid amplification-induced bias between clones. The emulsion was shaken at 40 rpm for 5 h at 37°C and then destabilized to combine all amplified phage. Amplification increased the titer from 10^6^ to 10^12^ PFU; on average, each library clone should be amplified by a factor of 10^6^. ssDNA was isolated from 10^11^ PFU.

The phage population from each condition was processed for deep sequencing as described below.

### Illumina sequencing

The steps for deep sequencing of phage libraries and analysis of the results were similar to those described in our previous report (21). In short, we isolated ssDNA from M13 phage using NaI/EtOH precipitation and purified it using phenol-chloroform extraction. The variable regions were isolated from the library and amplified by polymerase chain reaction (PCR) (25 ng of ssDNA) using barcoded primers (See Supplementary Scheme S1). We used 35 cycles of amplification because this protocol was suggested by Illumina (IL) and used by phage display researchers in at least three independent groups (18,40,41). The dsDNA PCR fragment corresponding to the expected size was purified by gel extraction. A total of 75 ng of the PCR fragment from each library was pooled together and processed for IL sequencing using the manufacturer’s protocols for end repair, adenylation, adapter ligation and PCR amplification of the product. The samples were sequenced on HiSeq IL using a 50-bp single-end run. FASTQ files were analyzed using custom MATLAB scripts (Supplementary Scheme S3). The software generated plain text-based lists of sequences and their abundances (Supplementary Scheme S2). These text files were used by MATLAB scripts to generate Figures 3–7 (see ‘Data Visualization’ section below). Raw FASTQ files (>10 Gb of data) are not included in this manuscript, but are available on request.

### Mathematical representation of sequence uniqueness and their abundance

A given list of sequences [s_1_, s_2_ … s_n_] can be conveniently represented as mathematical multisets, a set in which members can appear more than once (42). A multiset (S, m) is made of a S set of all unique sequences, and m is a vector, in which the m_i_ is a count of the sequence element S_i_. For more information about multiset notation and visualization techniques, please see Supplementary Scheme S3 and accompanying text.

### IL analysis

Sequences emanating from each amplification condition were identified using their respective barcodes (Supplementary Scheme S2). Abundances of the sequences and their quantities are described in Supplementary Figure S1. In short, ∼98% of the sequences could be mapped to a specific barcode. In the mapped sequences, 60% of the sequences contained all nucleotides with Phred Score >30. From these sequences, 80% contained nucleotides with (NNK)_n_ structure (where N is any nucleotide and K is G or T). We selected only sequences that had NNK structure and a Phred >30 for each nucleotide. We note that IL sequencing yielded both forward (F) and reverse (R) sequences originating from the (+) and (−) strand of the vector. The ratio of sequence abundances in F and R multisets varied from 40 to 60% (Supplementary Figure S1). In our processing, after removing non-NNK sequences and Phred <30 sequences, we observed significant overlap in sequence identity in F and R populations and similar sequence abundances in these populations (Supplementary Figures S14 and S15). We combined the multisets F = (F,f) and R = (R,r) using union definition: C_F∪__R_ = [F ∪ R, max(*f*,*r*)]. The union is the list of all unique sequence from either F or R, where the count of each sequence is equal to the maximum number of its appearances in F or R (the appearance was assigned 0, if the sequence was not present in one of the sets). In canonical bioinformatics terms, if f_ij_ is the number of forward reads for sequence i in library j and r_ij_ is the number of reverse reads, the union count k^∪^_ij_ is k^∪^_ij_ = max(f_ij_; r_ij_). In the combined multisets, we did not consider the sequences with a copy number *n* < 10 in our definition of parasites with the exception of analysis by ‘volcano plot’, which was supported by biological replicates (BR). Changing from the union to intersect-based processing, C_F∩R_ = [F ∩ R, max(*f*,*r*)], had little impact on the results of this manuscript because sequences with *n* > 10 were similar between F ∪ R and F ∩ R populations (see Supplementary Figures S14, S15 and S16, and ‘Discussion’ section in the main text).

### Ion torrent sequencing

We isolated ssDNA from M13 phage libraries using QIAprep Spin M13 kit (#27704). The isolated phage ssDNA (50 ng) was subjected to PCR amplification with primers flanking the variable region. To avoid a second round of PCR amplification, the primers contained Ion Torrent (IT) adapters at the 5′ ends. The concentration of PCR fragments that resulted from amplification of phage libraries was determined by analytical gel [2% (w/v) agarose gel in Tris Borate EDTA (TBE) buffer using a low molecular weight DNA ladder as a standard (NEB, #N3233S)]. dsDNA fragment (40 ng) from multiple PCR-amplified phage libraries were pooled together before running on E-gel. The band corresponding to the expected dsDNA product was purified on an E-gel SizeSelect 2% gel (Invitrogen, #G6610-02). The dsDNA fragments were extracted with RNAse-free water and the concentration determined by Qubit Fluorimeter (Invitrogen, #Q32851) using manufacturer’s protocol. The dsDNA fragments were ligated onto Ion Sphere Particles (ISPs) and amplified by emulsion PCR according to IT protocol. The concentration of ISPs with ligated dsDNA fragments after emulsion PCR was determined using Qubit Fluorimeter (Invitrogen) according to manufacturer’s protocol. The ISPs with ligated dsDNA fragments were enriched for and loaded on an Ion 316 chip. The sequencing was performed using an IT system (Life Technologies) with an Ion OneTouch 200 Template Kit. The FASTQ data from IT was processed by custom MatLab script that identified the barcodes, constant flanking residues, extracted the reads of the correct length (21-mer only) and correct (NNK)_7_ structure.

### Volcano plot

This plot identified sequences that increased significantly in frequency from the naïve library after amplification. As BR for the naïve library, we used eight separate instances of isolation and sequencing of naïve library (8 separate samples of 10^8^ PFU from naïve library, lot # 0061101, were processed and sequenced separately). We compared them with five BR of amplification (5 separate samples of 10^8^ PFU from the naïve library lot # 0061101, each amplified by a factor of 10^6^, and each was processed and sequenced separately). We normalized copy numbers by the total number of reads in each replicate and we considered all data that was observed either in the naïve or amplified populations. We did not remove the singleton population; furthermore, sequences not observed in a specific replicate were assigned a copy number of 0. Significance was assessed using one-tailed, unequal variance Student *t*-test. We also built a volcano plot using more rigorous statistics based on a negative binomial distribution and exact test with multiple testing correction (for details, see Supplementary Material S1–S5). Data from both plots were analyzed side by side (e.g. in comparison with MimoDB and non–peer-reviewed literature published on the Internet).

### Generation of stacked bars and scatterplots

Stacked bars, Venn diagrams and scatter plots in Figures 3–7 were generated by one MatLab script *command_center.m*, which contains a user-friendly graphic user interface (see Supplementary Material S4). We wrote the custom script to generate QQ-plots, volcano plots, histograms of ratios and complex scatterplots; the scripts are available as Supplementary Material under the names *MakeFigureXX.m* for various XX (e.g. script *MakeFigure5E.m*-generated plot from Figure 5E). Most raw files were not included in the Supplementary Material owing to their large size (17–130 Mb). The files are available at http://www.chem.ualberta.ca/∼derda/parasitepaper/

**Figure 2.**
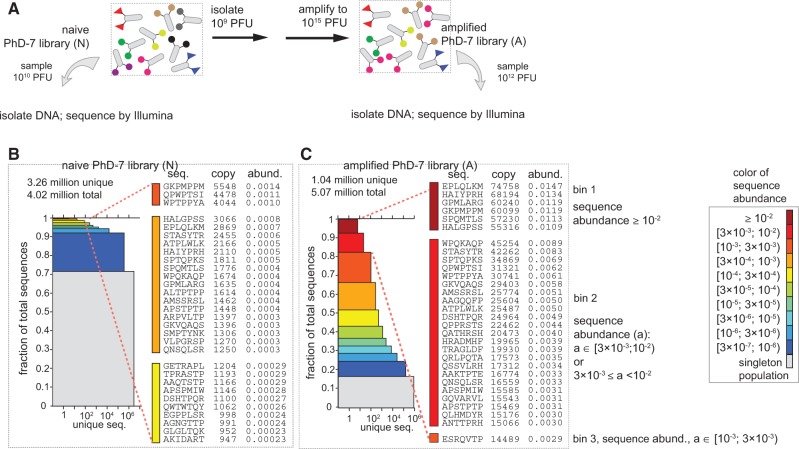
(**A**) We selected 10^9^ PFU from Ph.D.-7 library, amplified in bacteria, isolated the phage genome, amplified the library portion by PCR and obtained 4–5 million sequences using IL HiSeq. (**B** and **C**) To visualize all sequences, we generated a stacked bar in which each segment contains all sequences with specific abundance (color-coded); the width of each segment is equal to the number of unique sequences per segment. Before amplification (B) the majority of the clones in naïve library have low abundance. After amplification (C), ∼8% of the library is occupied by six sequences (crimson segment), ∼20% of the library is occupied by 35 sequences (red + crimson segments), etc.

To calculate the dimensions of the 2D stacked bars segments for the library of S^all^ total sequences, we converted copy numbers (N_i_) to sequence abundance as N_i_/sum(S^all^) and binned the sequences to approximately log-uniform bins (0.3 1], (0.1 0.3], (0.03 0.1], etc., where we assigned sequence *i* to bin (N_1_ N_2_] if N_1_ < N_i_ ≤ N_2_. In Figures 2 and 7 and Supplementary Figures S2, S10, S11, S14 and S15, each bin was represented by a segment of specific color. The height *h* and width *w* of the segment representing each bin was calculated as h^bin^ = S^bin^/S^all^, and w^bin^ = log_10_(U^bin^), where S^bin^ is the total number of sequences and U^bin^ the total number of unique sequences. Specifically, in Figure 2C as an example, the top crimson segment contains six unique peptides (U^crimson^ = 6) with abundance ≤0.03 and >0.01. These peptides constitute 8% of the library (S^crimson^/S^all^ = 0.08). The peptides in the bottom blue segment also constitute 8% of the library. This segment, however, contains 100 000 unique peptides (U^blue^ = 100 000). Each peptide has an abundance ≤0.0000003 and >0.0000001. Bottom gray segment represents the singleton population (sequences were observed only once).

### Web software for prospective identification of parasitic sequences

We provide an example of implementation as an open-source online script for use given a list of peptide sequences (http://chem-derda-web.chem.ualberta.ca/). The web application converts sequences provided by the user to a stacked bar (Figure 2B) and colors the segments according to their propensity to be ‘parasitic sequence’ using internally stored deep-sequencing data.

### Analysis Ph.D.-7, Ph.D.-12 and Ph.D.-C7C library screens

The literature data of phage display screens that used Ph.D.-7, Ph.D.-12 and Ph.D.-C7C libraries were extracted from the raw MimoDB 2.0 database. MimoDB is a database of all peptides identified by phage display screens (43). We used this database provided by Jian Huang, from which we extracted hits for each library (files are available in the Supplementary Material). The files were used by the *command_center* script to generate bar and scatterplots in Figure 6 and Supplementary Figure S11. (see Supplementary Materials and Methods for more details).

### Internet search for parasite sequences

We built a custom MatLab script *googlesearch.m*, available in the Supplementary Material, to streamline the Google™ search. A search URL was concatenated from '*https://www.google.ca/search?hl=en&as_q=*', ‘peptide sequence’ and ‘*&as_epq=&as_oq=peptide+++&as_eq=&as_nlo=&as_nhi=&lr=&cr=&as_qdr=all&as_sitesearch=&as_occt=any&safe=images&tbs=&as_filetype=&as_rights=*'. The HTML was loaded and parsed in Matlab to discard the results that contained no hits (∼90%); the remaining 10% were batch-loaded and inspected manually. On average, we were able to process 500 peptides in <30 min. The results of the search and URL of all identified references are available in the *allparasites.xls* file (Supplementary Material)

## RESULTS

### Identification of parasitic sequences using deep sequencing of naïve and amplified PhD-7 library

Our report focuses on the library of 7-mer peptides (Ph.D.-7™) because the reported diversity of the library (10^9^) approaches the theoretical diversity of (NNK)_7_ motifs (1.3 × 10^9^) and it covers most amino-acid diversity. To assess the diversity of the naïve library, we isolated DNA from 10^10^ PFU from the naïve Ph.D.-7 library (Figure 2A); this number should yield, on average, 10 copies of each available sequence, if the library was uniform. Sequencing of DNA by IL yielded 4 × 10^6^ reads (Figure 2B). Although sequence coverage was not complete, it was sufficient for our analysis here. If the naïve library contains 10^9^ sequences in equal abundances, the expected value of abundance of each sequence in a subsample of 4 × 10^6^ reads is 4 × 10^6^/10^9^ = 0.004. For this expected value, the Poisson probabilities to find a sequence with copy number 1, 2, 3 or 4 is 0.996, 0.002, 3 × 10^−^^5^ or 3 × 10^−^^9^, respectively. Over 99% of the population, thus, should have a single copy number (‘singleton population’). In 4 × 10^6^ reads, we expect at most one sequence with copy number strictly above three. In reality, we found that only 72% of the library contained a singleton population (gray segment, Figure 2B), 20% contained sequences with copy number of 2 or 3 (blue segment, Figure 2B) and 8% of the library had copy number of >3. Some sequences had a copy number >1000 (Figure 2B, list of top 30 sequences).

**Figure 3.**
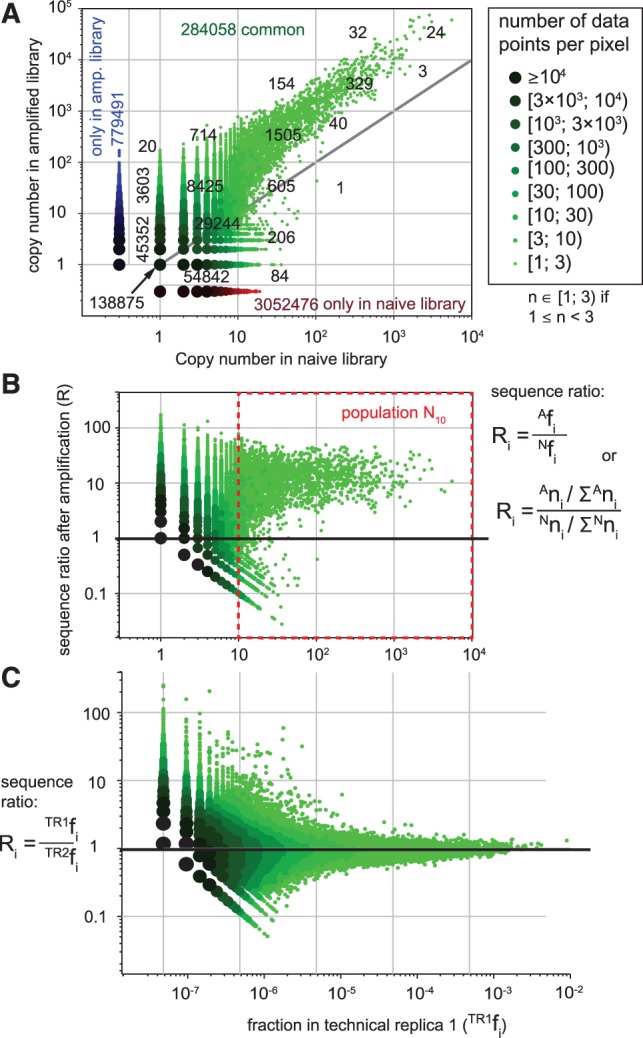
(**A**) Scatterplot describing naïve (N) and amplified (A) Ph.D.-7 library (condition 2, see ‘Materials and Methods’ section). Each dot is a unique sequence; multiple data at the same (x,y) coordinate are bigger, darker dots (see legend). Numbers represent the number of data points within each cell of the rectangular grid. Green data are observed both in N and A, while blue and red data are unique to N or A. (**B**) Ratio plot compares normalized ratio of each sequence between naïve and amplified library and copy number in naïve library. Copy number of many sequences present in the naïve library at copy number n_naïve_ >10 (red box, N_10_) increased during re-amplification. (**C**) The ratio plot similar to (B) comparing the same phage library samples by IL twice [data from reference (21)]. Distribution of the ratios of two technical replicates TR1 and TR2 is symmetric around 1.

We hypothesized that library members present at higher than theoretical abundance are the rapid-growing clones. Their number, thus, must increase if the library is re-amplified in bacteria. To validate this hypothesis, we amplified 10^9^ PFU from the naïve library in bacteria to yield 10^15^ PFU (expected amplification by a factor of 10^6^ for each clone) under amplification condition 2 (See ‘Materials and Methods’ section). Isolation of DNA from the amplified population and IL sequencing yielded ∼5 × 10^6^ reads. We observed that sequences that had high copy number in the naïve library N (e.g. GKPMPPM: copy number 5548, abundance 0.0014, Figure 2B) have been enriched in the amplified library A (GKPMPPM: copy number 60099, abundance 0.012, Figure 2C). Copy numbers of sequences in amplified libraries reached >50 000; this number, when normalized to total number of reads (5 × 10^6^), corresponds to 1% of the abundance in the library (sequences in the crimson segment of Figure 2C). Comparing N and A multisets by scatterplot (Figure 3A) and ratio plot (Figure 3B) traced the fate of all parasitic sequences during amplification. It suggested that most sequences with a copy number >10 in the naïve libraries have been enriched during re-amplification (Figure 3A and B). Previously, we have shown that IL sequencing of the same amplified population of phage yielded reproducible copy numbers (21). Figure 3C shows the ratio plot of re-sequencing data and suggests that increase in copy numbers in amplifications is not the result of sequencing bias. We sought to validate that the observed data are not the result of the biological variability in amplification or technical variability in sample preparation for deep sequencing.

### Variability of sequence abundances during phage amplification

**Figure 4.**
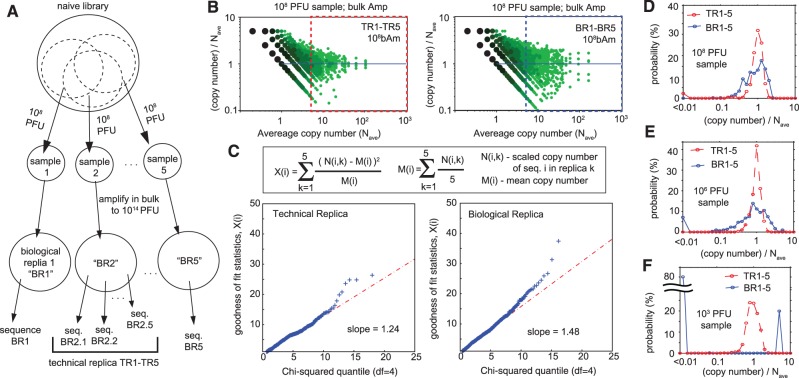
(**A**) Scheme describing generation of BR and TR. (**B**) Scatterplot of copy numbers in five replicates normalized by the mean copy number. (**C**) QQ-plots comparing goodness-of-fit statistics X(i) of scaled counts N(i,k), assuming Poisson distribution (44) and χ^2^ distribution with 4 degrees of freedom. Scaling factor was estimated as the total number of reads in library j divided by the average of the total read count across all libraries. The slopes of 1.25 and 1.5 suggested that the dispersion is 25% higher than Poisson for TR and 50% higher than Poisson for BR. Increase in BR is the result of the noise during PCR or re-amplification of phage in bacteria. The data deviates from the straight line because dispersion is not equal for all counts (confirmed by tagwise dispersion estimate for BR in Supplementary Figure S6). (**D** and **F**) Comparison of the distributions of the normalized copy numbers in BR and TR originating from different sample sizes. BR that start from 10^6^ PFU (**E**, blue line) have higher variance than BR that start from 10^8^ PFU (D), while BR that start from 10^3^ PFU are not reproducible; all TR are reproducible and have similar variance (red line).

Copy numbers in deep sequencing only approximate the true sequence abundance. Variability in copy numbers in re-sequencing of the same DNA samples could be modeled by Poisson distribution (44); variability in sequencing of closely related biological samples follows a Poisson distribution with Gaussian noise (45). Variability of the amplification process in phage libraries, however, has never been characterized. To this end, we analyzed multiple BR of phage amplification using lower cost (and lower throughput) IT sequencing. We estimated how naïve and amplified libraries would look at lower sequencing resolutions (Supplementary Figure S2). The analysis suggested that the high copy number sequences in amplified libraries should be readily identified from amplified libraries by IT. Most of the high copy number sequences visible in amplified libraries by IL were also identified by IT sequencing (Supplementary Figure S3). Figure 4A describes the sampling process: five BR originated from five independent samples of the library, 10^8^ PFU each. Every population of phage was amplified by a factor of 10^6^ in bacteria and sequenced independently. Additionally, we generated five technical replicates (TR) by isolating the DNA from the same amplified library five times and sequencing it separately. We examined how copy number in each scaled read count deviated from an average value across libraries, and indeed observed higher variance in BR than in TR (Figure 4B). We calculated the Pearson's cumulative test statistic from five replicates (Figure 4C) and compared it with a chi-square distribution with 4 degrees of freedom (44). A QQ-plot confirmed that copy numbers in TR and BR had a larger variance than would be predicted by a Poisson distribution, where the variance of TR and BR are ∼1.25 and 1.5 times larger than expected under a Poisson distribution (Figure 4C).

Our TR contained three sources of noise: (i) DNA isolation; (ii) PCR amplification and (iii) sequencing. Deviation from Poisson distribution caused by PCR re-amplification and re-sequencing has been observed previously (45). The BR contained (iv) variability in phage amplification and (v) variability in the composition of the initial sample. The latter increased as the sample size decreased from 10^8^ to 10^6^ PFU (Figure 4D and E). Decreasing the sample to 10^3^ PFU made all five BR completely irreproducible (no common sequences were observed among five BR, Figure 4F). In conclusion, when sample size is sufficiently large (here 10^8^ PFU), the biological variance is only 2× higher than technical variance and observable copy numbers are reproducible and normally distributed. Low-PFU samples are theoretically attractive because they could be sequenced with high coverage by medium-throughput sequencing; but for library with 10^9^ theoretical clones, repeated amplification starting from 10^3^ PFU yield misleading and irreproducible BR.

### Statistically significant definition of the fast-growing (parasite) sequences

**Figure 5.**
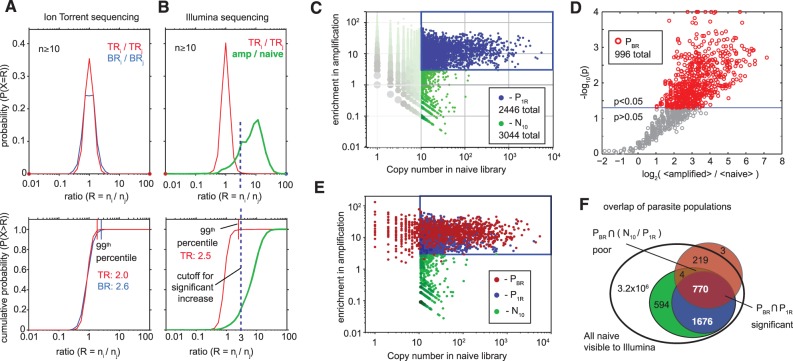
(**A**) Distribution and cumulative distributions of ratios observed between TR or BR described in Figure 4A and D. Less than 1% of sequences increased by >2.6-fold in BR. (**B**) Distribution of ratios in TR of amplified and naïve libraries from Figure 2C. Both A and B used reads with copy number >10. (**C**) The 99^th^ percentile of replicate in (A and B) suggested the use of 3-fold increase in n(amp)/n(naïve) ratio to define parasite populations, referred to as P_1R_. (**D**) More rigorous definition of parasite population, denoted as P_BR_, used five BR of the amplified population. Volcano plot highlights 996 sequences that increased significantly (*P* < 0.05) in amplification. Ninety-nine percent of sequences increase by >3-fold. (**E**) Mapping the P_BR_ population onto a parasite population defined by one replicate of IL Sequencing (P_1R_). Some sequences identified in P_1R_ have copy number <10 in naïve library, but all of them increase in amplification (as predicted by IL). (**F**) Venn diagram description of the overlap between naïve, P_10_, P_1R_ and P_BR_ populations.

Using multiple biological and TR, we established the limits of the variance in ratios of copy numbers in repeated amplification experiments. If deep-sequencing data were filtered to remove copy numbers <10, the 99^th^ percentile of the distribution of ratios was 2.4–3.0 in technical or BR on IL and IT platforms (Figure 5A and B). The deep sequencing data acquired by high-throughput hiSeq IL, thus, could be analyzed by these two criteria—n(naïve) > 10 and n(amp)/n(naïve) > 3—to define a population of parasites significantly enriched during the amplification process (Figure 5C). As this definition does not use true BR, only extrapolated variance, we call this population P_1R_ (parasites based on one replicate).

In lower-throughput methods, such as IT, significance based on cutoff in copy numbers is unreliable because few reads have n(naïve) >10 (Supplementary Figure S3). For IT, the significance of increase could be determined from *k* BR (here *k* = 5) generated by sampling and amplifying 10^8^ PFU and *m* re-sequencing instances of the naïve library (here *m* = 8). For the i^th^ sequence, we calculate the fold increase as f_i_ = 〈n_ik_(amp)〉/〈n_im_(naïve)〉 where 〈..〉 denotes averaging over replicates, and estimating the statistical significance t_i_ of this increase using one-sided unequal variance Student’s *t*-test. The resulting f_i_-t_i_ plot (‘volcano plot’) for ∼10^5^ sequences appears in Figure 5D (each dot is a unique sequence). We identified 996 parasites at a significance level of 5% and termed this population P_BR_ or ‘parasites based on biological replicates’. While P_BR_ originates from a different platform and a different type of statistical analysis, 80% of P_BR_ can be found in the P_1R_ population (Figure 5E). The remaining 20% of P_BR_ were found in the population with n(naïve) < 10, but the majority of these sequences (∼99%) exhibited an increase in copy number by IL sequencing [n(amp)/n(naïve) > 3], Figure 5E). Identification of a similar parasitic population from two separate sequencing platforms and two types of analysis confirmed that increase in ratio of copy numbers is neither the result of sequencing artifacts nor biological noise.

### Identification of the enriched (parasite) sequences using a negative binomial model

The 996 parasites in the previous section were identified based on statistics that relied on an incorrect assumption of normality as well as independent testing of significance for each parasite without correction for multiple testing. We aimed to check that these conclusions remain valid if we apply more rigorous statistical analysis. In the re-analysis of data, we accounted for three factors: (i) appropriate modeling of the counts using a negative binomial model, which allows for overdispersion when compared with Poisson distribution (Figure 4C); (ii) Benjamini–Hochberg (BH) correction for multiple testing (46), which was not accounted for in conventional *t*-test analysis and Volcano plot (Figure 5D); (iii) improved normalization of data across multiple replicates using the Trimmed Mean of M-values (TMM) normalization.(47) The integrated re-analysis of data was performed using Bioconductor package edgeR (48,49) (see Supplementary Section S1–S5 for R-code). We combined 10 replicates of Naïve library and 5 BR of library amplified from 10^8^ PFU (BR8) (Supplementary Figure S4). The edgeR analysis identified 606 parasites based on uncorrected *P*-values and 219 parasites after correction for multiple testing (Supplementary Figure S5 and S7). The overdispersion parameters estimated by edgeR were not constant but varied for different parasites (Supplementary Figure S6). The parasites defined by edgeR (designated as P_ER_ population) resided at the intersection of previously defined P_BR_ and P_1R_ populations. All definitions of parasites were similar for reads with high copy number, but P_ER_ parasites were scarce in reads with copy number <100 (Supplementary Figure S8B). The negative binomial model with TMM-normalization was designed for data that have relatively high copy numbers (e.g. RNA-seq data), and this algorithm tends to have weak detection power for low copy number reads (Andrea Rau, personal communication). In addition, the abundance of low copy number reads made the analysis sensitive to the model used for the estimation of dispersion parameters. For example, the DESeq Bioconductor package, which estimates per-sequence dispersions based on a local or parametric regression between means and dispersion estimates (50), produced significantly fewer enriched sequences: 294 without BH correction and 156 after BH correction.

### Parasitic sequences in the literature

**Figure 6.**
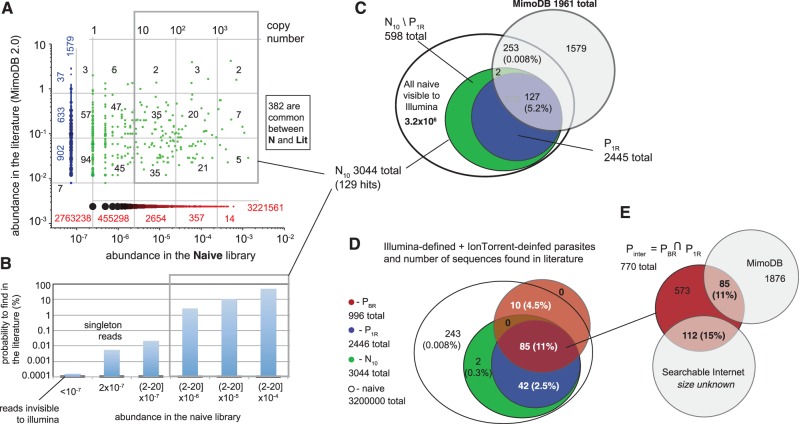
(**A**) Scatterplot comparing the abundance of sequences found in the literature (MimoDB database) and the naïve library sequenced by IL. Each dot is a unique sequence; multiple data at the same (x,y) coordinate are bigger, darker dots. Numbers represent the number of data points within each cell of the rectangular grid. Green data describes common sequences, while blue and red describe data unique to the MimoDB database or the naïve library. (**B**) Abundance of a sequence in the naïve library is correlated with the probability of finding this sequence in the literature. Abundance is reported as range: (2–20] means that abundance is >2 and ≤20. The second bar represents singleton reads; hence, abundance is not reported as range; the first bar represents the reads that were not found in the IL run. They are calculated as a difference between all possible 7-mer peptides and observed peptides (X7\IL). (**C**) Overlap between MimoDB and two putative parasite populations defined by IL. P_1R_ population (see Figure 5) has the most significant overlap with literature. The overlap is >1000-fold higher than overlap between MimoDB and 3000 random sequences, see Supplementary Figure S9). (**D**) Overlap between MimoDB and parasite populations defined by IL (P_1R_ and P_10_) and IT (P_BR_ from volcano plot, Figure 5). The P_BR_ ∩ P_1R_ population (crimson) has the highest overlap with the literature. (**E**) From 770 peptides in P_BR_ ∩ P_1R_ population, we found 85 in MimoDB; we performed an exhaustive Google search using 685 remaining peptides and found additional 112 peptides in the patent literature, published thesis work and peer-reviewed publications not yet included in MimoDB.

The hypothesis formulated in Figure 1 predicts that fast-growing sequences should be commonly identified during panning against any target. To test this hypothesis, we used MimoDB to extract sequences found in most peer-reviewed literature reports that used Ph.D.-7 library (Lit) to date (51). Six observations are important: (i) 382 out of 2000 Lit peptides could be identified in the entire Naïve library (Figure 6A). (ii) The ‘hit rate’—that is, the probability to find peptides in the naïve library—increased as we focused on subpopulations with higher copy numbers (Figure 6B). The ‘hit rate’ changed from 0.01% in the entire N to 4.3% in P_10_, in a subpopulation of ∼3000 peptide sequences with a copy number *n* > 10. (iii) From 129 literature hits in the P_10_ population, 127 resided in a parasite population P_1R_ identified from one round of IL sequencing (hit rate: 5.3%). (iv) Parasites defined by IT and BR P_BR_ contained 95 results from the literature (hit rate: 9.5%). (v) From 770 sequences in P_1R_ ∩ P_BR_ population, which contained parasites found by both sequencing platforms, 85 were found in the literature (hit rate: 11%). (vi) From the focused population of 219 hits defined by EdgeR (P_ER_), 48 were in the literature (hit rate: 22%) (Supplementary Figure S8). The simultaneous increase in hit rate and decrease in the number of literature hits suggests that parasite population P_ER_ is more specific than P_BR_ (9.5 versus 22% hit rate) but less sensitive (e.g. 95 versus 50 hits in literature hits). While we cannot estimate the exact number, it is possible that at least a few of the 45 ‘missed hits’ are true ‘false negatives’. For example, peptide NQDVPLF identified in P_BR_ but not in P_ER_ has a relatively high copy number in naïve library (58 copies, 0.002% abundance) and it has been identified as a ligand for at least seven unrelated targets: 16S RNA (52), chromatin high mobility group protein 1 (HMGB1) (53), kidney (*in vivo* panning) (54), human synovial B cell hybridoma ELB13/3-56 (55) antibody against *Neisseria meningitidis* group(56) and cyclodextrin (patent) and 001 face of nacreous aragonite (Ph.D. thesis). Other potential ‘false negative’ peptides missed in P_ER_ are peptides that bind to more than one target (according to Internet search in peer-reviewed and other literature): examples are SPPQSRA (mesenchymal cells and antibody 5F1), KQTLPSA (HUVEC cells and oocytes) and AVPRASF (lipopolyscharide and S16 RNA) (complete list is provided in allparasites.xls in the Supplementary Material).

Statistical significance of the observations above can be validated using a series of null hypotheses (H_0_). To test observation (i) the null hypothesis was: ‘For a peptide found in the literature, the probability for it to appear in our naïve library is no different from the probability for it to appear in a random library of the same size’, where, by ‘random library of the same size’, we mean a library of 3.2 million peptides that were chosen at random from all possible 7-mer peptides encoded NNK codons. The problem can be solved using Fisher's exact test (Simon Anders, personal communication), by using amino acid composition of each literature hit to calculate its exact probability to be found in a (NNK)_7_-encoded library of peptides. As an alternative, we used bootstrapping simulation, in which we generated random uniform libraries of 3.2 × 10^6^ (NNK)_7_ encoded peptides *in silico* and calculated Lit ∩ Rnd^3200000^. As expected from the Fisher’s test, the simulated values of intersection between Lit ∩Rnd^3200000^ followed Poisson statistics with an expectation value of 15 (Supplementary Figure S9A). The probability (*P*) to observe ≥382 common sequences was *P *≪ e^−^^382^. This result suggested that the much larger observed overlap between Lit ∩ N is not due to chance, but may instead be the result of diversity collapse via similar forces. Testing a general hypothesis for sample size *m* assessed the expected overlap between the literature and any sample Lit ∩Rnd^m^ (Supplementary Figure S9F). For example, Rnd^770^ had the same size as the ‘focused parasite population’ (P_1R_ ∩ P_BR_, Figure 5F). The probability to find a population of 770 random peptides that contained even one literature hit was 0.4% (1 in 250 populations contained one literature ‘hit’, the rest contained none). It was highly improbable (*P *≪ e^−^^85^) to ‘guess’ a population of 770 peptides that contained 85 sequences from the literature. Observations (ii) through (iv) could also be tested as another hypothesis: ‘parasites are a random subpopulation of naïve library’. Specifically, for (ii) H_0_:(Lit ∩ N^3000^) = (Lit ∩ N_10_) = 382 (For a peptide found in the literature, the probability for it to appear the specific list of 3000 parasites (N_10_) is no different from the probability for it to appear in a random subset of 3000 sequences from the naïve library (designated as N^3000^). We generated N^3000^ libraries by random sampling of the N library and observed that Lit ∩ N^3000^ followed Poisson distribution with an expectation value of 0.4. The probability to observe overlap of 130 peptides was *P *≪ e^−^^130^ (Supplementary Figure S9B). It is therefore essentially impossible to ‘guess the parasite sequences at random’ from a sequenced set.

To provide additional ‘replicates’ for the literature search experiment, we selected 770 peptides from the putative parasite population (P_TR_ ∩ P_BR_), eliminated 85 peptides found in MimoDB and searched for the remaining 685 peptide on the open Web using Google (see ‘Materials and Methods’ section). Interestingly, we found 112 matching peptides in various peer-reviewed and non-reviewed publications (Figure 6E). Specifically, 33 originated from PubMed-indexed peer-reviewed publications, 15 were from published theses and the rest were from patent literature. All publications used the Ph.D.-7 library. References to all publications are available in the Supplementary Material. The 197 peptides found in a small 770-peptide population (P_TR_ ∩ P_BR_) doubled the discovery rate from 11% in MimoDB to 26% in the entire Internet (which includes MimoDB) (Supplementary Figure S8). We repeated the same search for P_1R_, P_TR_, P_BR_ and P_ER_ populations. Focused population P_ER_, which had 22% discovery rate in MimoDB, had 44% rate in the entire Internet. Populations N_10_ and P_1R_, which has a low discovery rate in MimoDB (4.3 and 5.1%), also doubled their rate in the ‘expanded search’ (8.9 and 12%). The same trend was observed in ‘negative control populations’, which were depleted of statistically significant peptides (Supplementary Figure S8).

From the size of the MimoDB database (∼2000 peptides) and the observed trends in discovery rates, we extrapolated the size of the expanded database as 2× MimoDB (∼4000). We thus estimated that every 20^th^ peptide ever reported in the literature originates from a subset of parasite peptides that constitute <10^−^^7^ of the available diversity. (We believe that there is a correlation between the lot number and the probability to identify a parasite. Unfortunately, it was impossible to map the lot origin of the libraries used in the literature because few publications report the lot number).

Some parasitic sequences we identified have been already characterized. Noren and coworkers identified that the HAIPYRH sequence is associated with phages that have mutations in the regulatory regions (37). This sequence has a copy number of >2000 in the naïve library and >68 000 in the amplified library (Figure 2B and C). This sequence appeared in screens against 13 unrelated targets (51), and has been confirmed as a weak binder for many targets. Other sequences have similar properties: GETRAPL (#21 in Figure 2C) was found in 4 independent screens; 6 independent screens identified sequence SILPYPY and 11 screens identified LPLTPLP (see *allparasites.xls,* Supplementary Material) (51,57). Sequences such as EPLQLKM (#1 in Figure 3D) have been identified in over six screens (58–60), annotated in databases and flagged as ‘suspicious’. Other sequences, such as sequence #8 STASYTR, have not been annotated in any databases yet, but it has been found in two published screens (61,62) and our own unpublished results. The parasite population has no common sequence motif. Aside from the small bias to Pro and Ser/Thr amino acids, we could not detect any sequence similarity in ‘parasites’. The sequences did not correlate with motifs that occur owing to nonspecific binding to polystyrene (4). The designation ‘parasite’ is different from ‘nonspecific binder’. In many publications, the binding properties of these sequences have been confirmed to be in the micromolar range. These observations confirm that the parasitic sequences are selected because they have both target binding capacity and high amplification rate (in line with our prediction in Figure 1).

### Bypassing selection of parasite sequences

**Figure 7.**
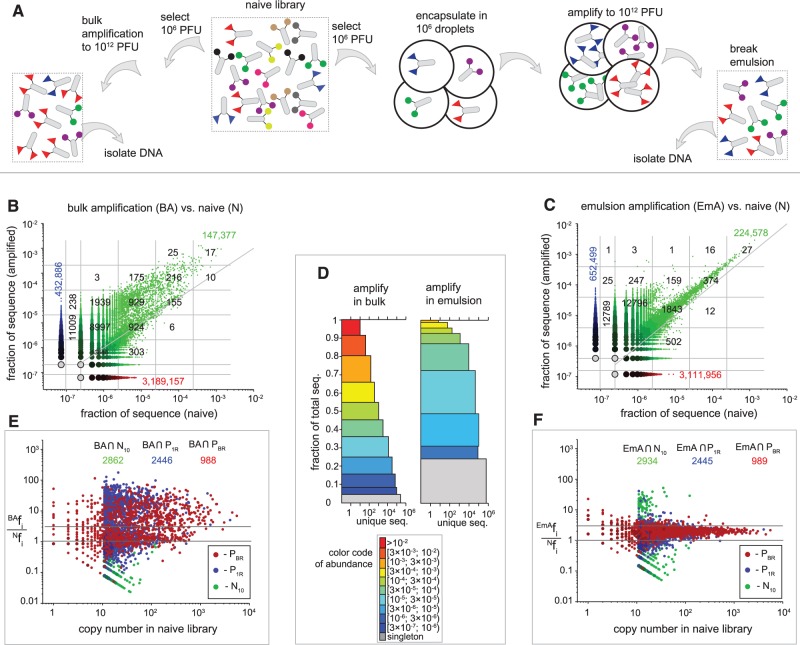
(**A**) Scheme of the amplification of 10^6^ PFU taken from Ph.D.-7 naïve library. Amplification was performed either in bulk or emulsion (as described in Conditions 1 and 3 in the ‘Materials and Methods’ section). (**B**) Bulk amplification or ‘BA’ shows significant enrichment of parasitic sequences when compared with emulsion amplification ‘EmA’ (**C**). (**D**) The sequences with high abundance (f_i_ > 10^−4^, orange-red segments) constitute ∼35% of the population after bulk amplification; these highly abundant sequences largely constitute <1% of the emulsion-amplified library (**E** and **F**). We monitored the fate of parasites (P_BR_ and P_1R_ populations). Both parasite populations are enriched during BA (E). (F) In EmA, the majority of the clones from the parasite populations increased by <3 (within the 99% confidence interval, as defined in Figure 5A).

Enrichment of parasites occurs owing to competition between phage clones during amplification in bacteria (Figure 1B). If competition between clones could be avoided, emergence of ‘parasites’ could be suppressed. Previously, we developed a technology to perform uniform amplifications in emulsions. We demonstrated that emulsions can be used to amplify a mixture of fast- and slow-growing phage clones uniformly (38,39). Here we demonstrate that emulsion amplification can bypass the biased overselection of parasitic sequences from large libraries. We have previously demonstrated that this technique is well-suited for amplification of 10^6^ PFU (38); we also observed that amplification experiments based on samples of 10^6^ PFU yields reproducible, albeit noisy, BR (Figure 4E). We selected 10^6^ PFU from the naïve library and amplified them to 10^12^ copies using bulk or emulsion amplification (Figure 7A) (for details, see conditions 1 and 3 in the ‘Materials and Methods’ section). The library after bulk amplification of 10^6^ PFU (Figure 7B and D) was similar to the library after bulk amplification of the entire 10^9^-scale library (Figures 2C and 3A). It contained the same parasitic sequences and >50% of them have been enriched beyond the variance of BR (>3-fold, Figure 7E); small deviations originated from a limited sampling in a 10^6^ PFU set. In contrast, the emulsion amplification maintained the abundance of the sequences (Figure 7C). The abundance of high copy number clones in the phage library amplified in emulsion was suppressed (Figure 7D). The abundance of the majority of the parasitic sequences from P_1R_ and P_TR_ populations remained within the variance of the BR. Their ratio increased by <3-fold (Figure 7F).

We emphasize that the use of emulsion amplification cannot fix the skewed diversity already present in the naïve libraries; it can maintain this diversity and minimize any further selection of fast-growing clones. We have used emulsion amplification in selection to show that such selection allows identification of sequences that cannot be identified by conventional phage display (red x in Figure 1C). These results, however, extend beyond the scope of this manuscript and they will be presented elsewhere.

### Other libraries

We observed similar results to those described above in other libraries: in Ph.D.-C7C (Supplementary Figure S10A and B) and Ph.D.-12 (Supplementary Figure S11A and B); namely, the diversity in naïve libraries was skewed, and it collapsed on re-amplification. We used these libraries to demonstrate that emulsion amplification is reproducible. The collapse of diversity in PhD-C7C and PhD-12 libraries was mitigated by emulsion amplification (Supplementary Figures S10C–G and S11C–G). We anticipate that the diversity of other phage libraries could be maintained by this method.

We propose that it should be possible to map parasitic sequences in other libraries using two simple steps. If diversity of the library is 10^k^ for some k > 1: (i) isolate the DNA from ∼10^k^ clones in the naïve library and sequence them to obtain several replicates of the naïve library (N). (ii) Amplify separate samples of at least 10^k^^−^^1^ clones from the naïve library by factor of 10^6^ and sequence them to get amplified libraries (A). Then, compare multisets A and N using statistical analysis (e.g. similar to volcano plot in Figure 5) to identify parasitic populations. We strongly believe that performing prospective identification of parasitic populations will be critical for selecting functional sequences from these libraries. This identification should become a standard protocol/practice for the researchers using these libraries, as well as commercial providers of these libraries. Both high-throughput methods like IL HiSeq and lower-throughput technique like IT could provide statistically significant results with high predictive power.

### Effect of sequencing errors on identification of parasites

Deep sequencing methods have an error rate of ≥1%. Our prospective identification by deep sequencing inevitably contained some false-positive reads (incorrectly interpreted) or false-negative reads (censored by sequencing). Distribution of Hamming distances in the library suggested that sequencing results contain a large number of point mutations (Supplementary Figure S12). Point mutations are not the result of phage amplification, and they originate from PCR or sequencing errors (63). For every abundant sequence, we identified a large number of mutants (MUT) in the library (Supplementary Figure S13A and B). Their abundance was 1–5% of the parent sequence (Supplementary Figure S13C). We developed an algorithm that tagged and removed MUT errors (Supplementary Figure S14) to create mutation-free (MUT^−^) libraries with a normalized Hamming distance profile (Supplementary Figure S12). The other known source of error is formation of hairpins during sequencing (64); it can change NNK structure (NNM errors) and skew the copy numbers in forward (F) versus reverse (R) reads (Supplementary Figures S14 and S15). We observed that in libraries made by intersection (F∩R) instead of union (F ∪ R) of reads, MUT and NNM errors were reduced but not eliminated (Supplementary Figures S14 and S15).

Our standard processing of sequencing data could be designated as (F ∪ R, MUT^+^, NNM^−^) (union of F and R reads, MUT were not removed, NNM sequences were removed). The entire manuscript could be re-analyzed using more stringent processing such as (F ∪ R, MUT^−^, NNM^−^) or (F∩R, MUT^−^, NNM^−^). This processing changed the apparent size of the libraries from 3.2 million for Naïve(F ∪ R, MUT^+^, NNM^−^) to 260,000 for Naïve(F∩R, MUT^−^, NNM^−^). The major conclusions of the article, however, were largely unchanged. The size of the parasite population and the number of sequences identified in the literature varied by ∼5% (Supplementary Figure S16). We observed an increase in copy number in bulk amplification and no increase in emulsion amplification (Supplementary Figure S16). Even the most stringent populations, such as (P_TR_ ∩ P_BR_) defined by multiple BR on two different sequencing platforms, could contain a few erroneous sequences. Still, we believe the method described here provides one of the most rigorous ways for the prospective identification of parasite sequences. The errors could be further decreased with advances in deep-sequencing techniques and improved error-analysis algorithms.

## DISCUSSION

For libraries made from 10^9^ transformants of randomized DNA vectors, the expected abundance of each sequence is 0.0000001% (65). However, our data indicates that as the DNA is translated and the naïve library is produced in bacteria, the abundance of parasitic sequences rose from 0.0000001 to >0.01% (over five orders of magnitude). Additional amplification of this library in bacteria increases the abundance of parasites to 1%. To our knowledge, this is the first time naïve libraries have been characterized at this level. The analysis of diversity as a result of amplification provides an explanation to several problems commonly observed in the phage display literature: (i) the majority of published screens could identify only a small number of binding clones; (ii) binding ability of phage rarely correlates with its abundance in the screen; (iii) screens against targets with multiple binding sites (cells and tissues) identify only a few hits. These observations were summarized in several recent reviews (4,23). To explain these observations, we proposed a 2D selection model (23), which describes how phage display selection and amplification drive collapse of diversity and lead to identification of only a subset of binding sequences (Figure 1). Deep sequencing data presented in this report strengthens this model.

Loss of useful binding clones cannot be mitigated by improved selection procedures: if multiple binders have an equal selection pressure in binding (equal K_d_) (66–68) and have unequal selection pressures in amplification (different phage propagation rates), the ‘slow growing’ binder always disappears from the selection and the ‘parasite’ is always selected. Such loss presents no problem if the screen aims to identify only one lead. Loss of binders, however, precludes simultaneous identification of ligands for multisite targets, such as mixtures of antibodies, and surfaces of cells and tissues. To select diverse sequences for these targets, one must reengineer amplification [e.g. use emulsion amplification (39)] or avoid amplification entirely and use deep sequencing to run selections without amplification (19). We note that for some targets, the properties of the sequence that generate stronger binding could be identical to those that enhance amplification. Such a possibility has been proposed for peptide libraries (22).

### Parasites and censored clones

Makowski and coworkers, among others, introduced the term ‘censorship’ to describe that some sequences are improbable to find in the library (22). They linked censorship to a specific pattern of amino acids at specific positions and they hypothesized that censored sequences displayed on phage inhibit infection and production of phage. Makowski also attempted to predict fast-growing sequences using the same positional abundance algorithm (22). Our report uncovers ‘parasites’, which do not have a specific amino-acid sequence. Their high abundance cannot be predicted from positional abundance of amino acids. For example, if positional abundance was important, most of the point mutants of the parasites should have high copy numbers as well (this hypothesis could be easily rejected by searching for any mutants of sequences in Figure 3C and D, see Supplementary Figure S13). The biological mechanism that makes some sequences ‘parasitic’ is already known: they emerge due to mutation in the regulatory region of the phage genome (37). This mechanism has been verified only for one parasitic clone HAIYPRH but it is possible that emergence of other parasites occur owing to a similar mechanism. Since the displayed sequence is not related to mutation in the regulatory region, it might not be possible to predict parasitic sequences. Instead, parasites have to be mapped prospectively for each batch of the produced library by sequencing a portion of the naïve and amplified library.

Smith and coworkers predicted the existence of ‘parasites’ but they hypothesized that the incidence of mutations that yield parasitic clones are rare and such mutations occur only after serial amplification (36). Our large-scale sequencing suggested the opposite: parasitic clones exist in the library immediately after generation; however, they become visible to small-scale sequencing only on serial re-amplification of the library. Deep-sequencing and appropriate statistical analysis could identify these parasites directly in naïve libraries using only one round of amplification.

### Prospective mapping of parasitic clones in all libraries

Our analysis of parasitic clones in this report is based on one lot of the phage library. NEB produced and sold >10 independent lots of their phage libraries (NEB, personal communication). As these lots could contain different sequences, our analysis does not contain all possible parasitic clones. This fact could explain the incomplete overlap of ‘parasitic clones’ with literature clones in Figure 6. Sequencing of all lots of all libraries produced to date could provide a powerful bioinformatics resource for analysis of past and future phage screens. Importantly, this sequencing could be completed using only 1–2 deep-sequencing runs of pooled libraries tagged by barcoded primers (21,41)

The examples presented here were related to peptide libraries identified via phage display. Identical steps can be used to analyze polypeptide libraries from other screens (e.g. RNA-, DNA-, ribosome-, bacteria- or yeast-display) and RNA/DNA aptamers. The molecular mechanisms that generate ‘parasitic’ sequences in RNA or DNA libraries (69,70) are different from the mechanism that leads to emergence of parasitic phage; the phenotypic outcome—enrichment in amplification—can be readily detected by deep sequencing. The online version of our visualization software can be expanded to allow for linking to existing databases that contain peptide or nucleotide sequences. We anticipate that the analysis techniques described in this report will improve analysis of selection and amplification from all genetically encoded libraries.

### Emulsion amplification and generation of parasite-free libraries

We believe that it should be possible to use emulsion amplification to repair the collapse of diversity that occurs during the generation of libraries in bacteria. The transformation of bacteria in emulsions has been reported (71,72). Large-scale emulsion-generation techniques to produce 10^8^–10^9^ droplets are also known (73). This large-scale transformation-in-emulsion could be used to generate naïve libraries with uniform sequence diversity. Due to rapid development of techniques for generation of monodisperse emulsions and their popularization in biotechnology (74), we anticipate that such capabilities could be achieved in a few years.

## SUPPLEMENTARY DATA

Supplementary Data are available at NAR Online, including [47–50].
